# Design and testing of ultrasound probe adapters for a robotic imaging platform

**DOI:** 10.1038/s41598-024-55480-0

**Published:** 2024-03-01

**Authors:** Krysta-Lynn Amezcua, James Collier, Michael Lopez, Sofia I. Hernandez Torres, Austin Ruiz, Rachel Gathright, Eric J. Snider

**Affiliations:** Organ Support and Automation Technologies Group, U.S. Army Institute of Surgical Research, JBSA Fort Sam Houston, San Antonio, TX 78234 USA

**Keywords:** Biomedical engineering, Mechanical engineering

## Abstract

Medical imaging-based triage is a critical tool for emergency medicine in both civilian and military settings. Ultrasound imaging can be used to rapidly identify free fluid in abdominal and thoracic cavities which could necessitate immediate surgical intervention. However, proper ultrasound image capture requires a skilled ultrasonography technician who is likely unavailable at the point of injury where resources are limited. Instead, robotics and computer vision technology can simplify image acquisition. As a first step towards this larger goal, here, we focus on the development of prototypes for ultrasound probe securement using a robotics platform. The ability of four probe adapter technologies to precisely capture images at anatomical locations, repeatedly, and with different ultrasound transducer types were evaluated across more than five scoring criteria. Testing demonstrated two of the adapters outperformed the traditional robot gripper and manual image capture, with a compact, rotating design compatible with wireless imaging technology being most suitable for use at the point of injury. Next steps will integrate the robotic platform with computer vision and deep learning image interpretation models to automate image capture and diagnosis. This will lower the skill threshold needed for medical imaging-based triage, enabling this procedure to be available at or near the point of injury.

## Introduction

For emergency medicine, triage is a fundamental step for identifying injury severity and prioritize use of resources when treating casualties. This is especially critical when resources are constrained, such as in remote medical situations faced in rural areas or in military medicine. While physical examinations and basic vital signs are frequently used for high level casualty triage, medical imaging can provide a higher granularity for triage which will be essential on the future battlefield. A wide range of clinical imaging options are available in civilian settings for routine emergency medicine, however, medical image capabilities at or near the point of injury are often limited, rendering ultrasound (US) the most convenient imaging platform, due to its small size, portability, and low power requirements^1^.

The most commonly used triage examination with US imaging is the Extended Focused Assessment with Sonography in Trauma (eFAST) protocol that quickly scans the thoracic and abdominal cavities for the presence of free fluid in the peritoneal, pericardial, and pleural spaces^2,3^. Air in the pleural space indicates pneumothorax, which must be treated before it worsens into a potentially fatal tension pneumothorax injury^4^. Fluid presence in the thoracic or peritoneal cavity indicates a hemorrhagic injury either internal or from a traumatic wound to the torso that may be actively worsening. Thus, a positive diagnosis on an eFAST exam often requires immediate surgical intervention which in remote medical scenarios will require immediate medical evacuation.

However, an eFAST exam is only as valuable as the quality of the imaging and the skill level of the clinician interpreting them, therefore performing exams accurately can be exceptionally challenging for inexperienced operators during high-stress, mass-casualty situations. To acquire accurate information from each scan point, the transducer must be properly positioned so that relevant anatomical information is visible in the ultrasound image. Furthermore, interpretation of ultrasound images requires highly trained personnel such as a skilled radiologist, who are likely not readily available at or near the point of injury. As a result, automation strategies for improving image acquisition and interpretation are critical for making ultrasound image-based triage for the injured warfighter accessible at or near the point of injury.

Image interpretation automation can be aided by artificial intelligent (AI) models tuned to identify abnormalities in ultrasound images. We have extensively evaluated AI models for various US applications including eFAST^5–8^, and it is the subject of many active research efforts^9–11^. In this work, we will focus on the image acquisition side of the ultrasound automation challenge.

Imaging automation utilizes robotic systems to hold the transducer while capturing ultrasound images. At a lower automation level, the robot can be remotely controlled for capturing images without the operator needing to be at the scan site^12,13^. This has the benefit of allowing a skilled operator to continue image acquisition, but it still requires human input throughout the entire scan. Furthermore, latency delays become a challenge as the distance between the operator and robot expands^14,15^. This is further complicated for remote emergency medicine situations where remote communication may not be feasible. Remote﻿ robotic imaging has been used for obstetric^16^, cardiovascular^17^, and abdominal^18^ imaging applications.

At a higher automation level, progress has been made toward fully autonomous image capture through the integration of mechanisms to account for force applied to the subject, whether it be through force sensors or spring mechanisms to regulate application force^19–21^. These mechanisms have been successfully integrated with different robotic platforms to ensure no harm is done to the subject during scanning and that sufficient force is applied for ultrasound image capture.

The next step in automated image capture is generating robotic movements to properly capture images. This is most often done offline by reference tissue phantoms or a camera attached to the robot allowing for an operator to precisely pinpoint where the robot should move and in what order to properly collect images^22,23^. This can be done in real-time instead, using computer vision AI to identify scan points based on subject anatomical landmarks^24,25^. However, computer vision alone cannot acquire proper US images due to the need for precise probe angling and positioning. Instead, more advanced “ultrasound visual servo-ing” techniques are needed which utilize ultrasound image feedback to position the robot properly for image capture^12^. Approaches have used reinforcement learning to try and learn how an experienced sonographer captures images as well as convolutional neural networks for assessing image stability or object tracking.

For designing a robotic system for eFAST assessment, a few additional challenges must be considered. First, as this is an emergency medicine triage tool, speed of the examination is critical. An average eFAST exam can take approximately 4 minutes^26^, so robotic image capture must be comparable to this. This will require more rapid decision making by the image capture setup than may be required for many other automation applications being considered. Second, the thoracic scan points traditionally use a linear ultrasound probe, while abdominal scan points use a curvilinear probe type, requiring the imaging system to be able to swap probes automatically while maintaining image capture accuracy.

As a first step toward automating eFAST image acquisition, in this effort we first focus on methods for securing an US probe to a robotic imaging platform. Purpose-built adapters are needed for accommodating the unique design considerations specific to eFAST image capture. Specifically, we evaluate four different US probe holder adapters paired with a Universal Robot arm to determine if the platform can be configured optimally for rapid, accurate, and versatile ultrasound image capture that conducting an eFAST examination will necessitate.

## Methods

### Overview of universal robotic platform

**Figure 1:**
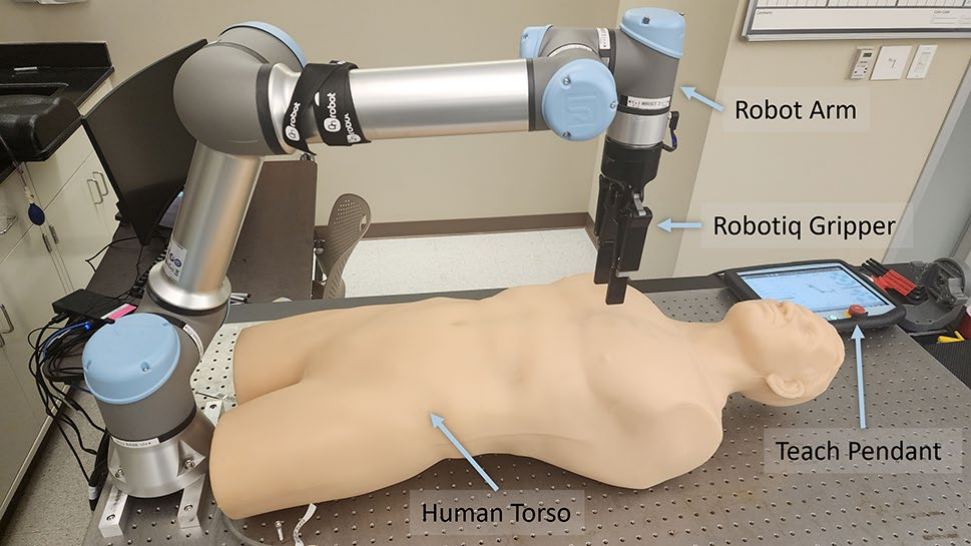
Diagram showing robotic arm setup for ultrasound imaging. The UR5e (‘Robot Arm’) is table-mounted and positioned over a torso phantom (‘Human Torso’) and equipped with an end-adapter (Robotiq Gripper is shown). Robotic motion control is programmed via the Teach Pendant.

We used a Universal Robot 5e (Universal Robots, Odense, Denmark) for attachment of various probe holder designs and movement of an ultrasound probe to acquire proper images (Fig. 1). The UR5e system has a maximum payload of 5 kg and 6 degrees of motion freedom, allowing for image capture at each of the various eFAST scan points. UR5e motion is programmed using on-board control capabilities via the Teach Pendant. Each probe adapter design (see section "Robotic probe holder designs") integrates with the UR5e, allowing the robot to hold an US transducer for performing the eFAST exam. A program was developed which provided UR5e control over the positioning of the probe using force and torque feedback at the end-applicator obtained through an integrated sensor (Robotiq, Lévis, Quebec, Canada).

### Robotic probe holder designs

Probe adapters were developed with several key design constraints. The adapters needed to ensure contact between the US probes and the subject, considering the range of motion of the robotic arm and other limitations such as length of the cord for standard ultrasound systems. Therefore, compact designs were prioritized. The probe adapter prototypes also required the ability to change between at least two transducers that are commonly needed to complete an eFAST exam—linear and curvilinear probes. The higher-frequency linear probe is used for thoracic imaging as the key structures are shallower while the lower-frequency curvilinear probe allows for visualizing deeper abdominal organs. This design feature would allow for the appropriate probe to be selected as necessary for each specific scan site. Therefore, modular and self-contained designs which addressed this need for probe exchange were considered as well. Additionally, considering the robotic platform application of medical imaging-triage, designs needed to account for reliability and time of probe exchange to decrease the time needed to perform the eFAST procedure. Since navigation to each scan point would be controlled by the robotic arm, duration of the exam would be most affected by the time required to swap probes. Finally, the probe adapter designs needed to apply force to the subject in a reliable manner which would allow for clear image capture at multiple scan locations. Therefore, designs needed to securely hold the probe in place under repeatedly applied forces.

Four different US probe holders were evaluated based on these design criteria. Three purpose-built prototype designs were compared against a fourth commercially available gripper, the 2F-140 adaptive gripper (Robotiq, Lévis, Quebec, Canada). Using this end-adapter’s adjustable grip width, it could be manipulated to hold any ultrasound probe for testing.

Each custom probe end-adapter design incorporated a probe swapping mechanism for ease of performing a full eFAST exam. The probe end-designs were designed to fit probe models from a Terason US (Terason, Burlington, MA, USA) system. One probe holder design was fit around a dual-sided wireless US probe, Vscan Air™ CL, (GE HealthCare Technologies, Inc., Chicago, IL, USA). However, to avoid damaging US probes during preliminary testing of the robotic imaging platform, a mock US probe of identical shape and size was cast with polyurethane (Clear Flex 50, Smooth-On, Easton, PA, USA) after an inverse mold was made using silicone rubber (Eco Flex 00–31 Near Clear, Smooth-On, Easton, PA, USA). In addition, each custom-design utilized a mold of the precise geometrical shape around the US probe using an epoxy resin (Smooth-On, Easton, PA, USA) so that the probe adapters could hold a more regular, defined geometry.

#### Gear design

**Figure 2:**
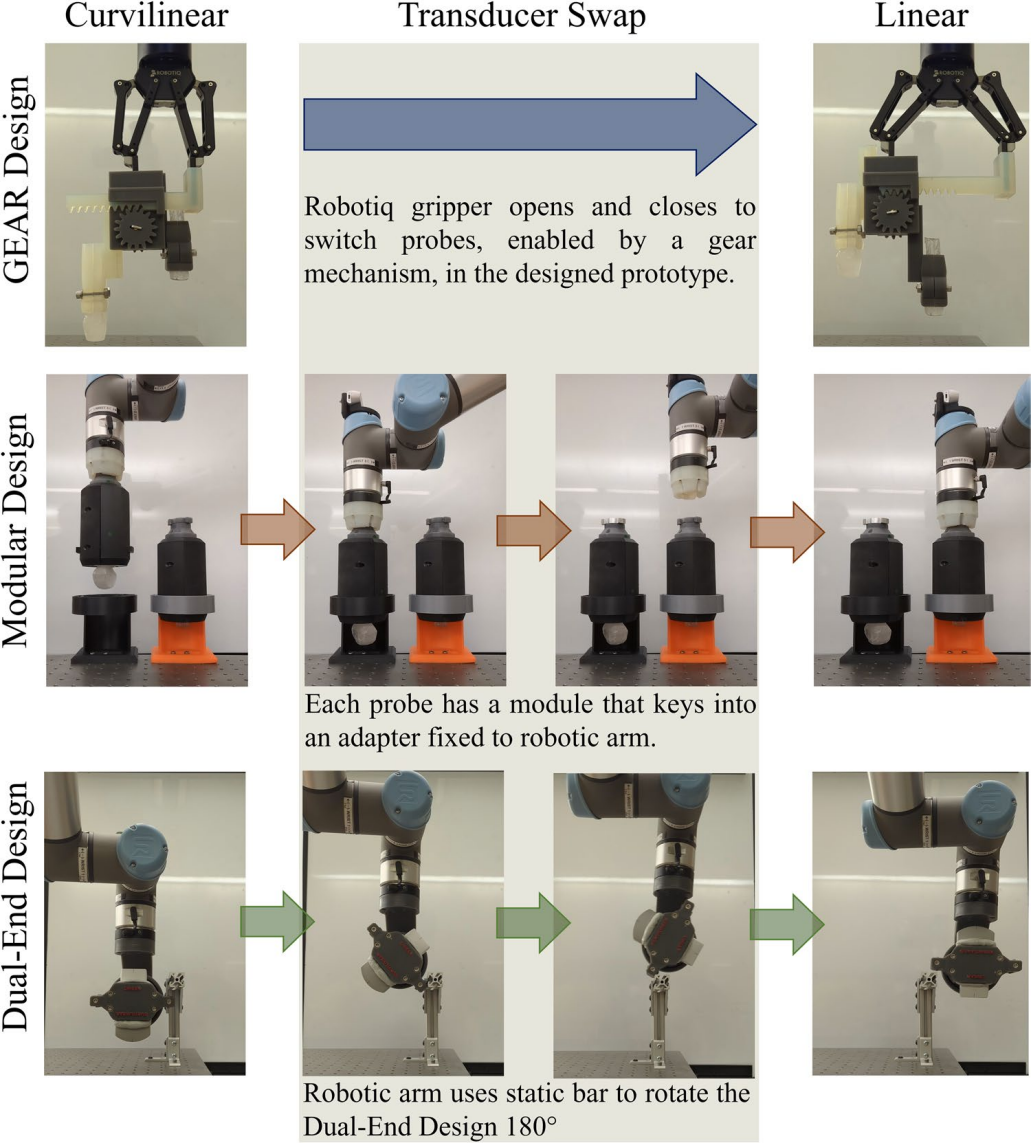
Comparison of each probe adapter design and probe swap mechanism. Three different designs are shown: Gear design (Row 1), Modular Design (Row 2), and Dual-End Design (Row 3). For each, the mechanism for probe swapping is shown from curvilinear (left) to linear (right) transducer. Intermediate images are shown when necessary to better display the probe swap technique.

The Gear Design was devised as a robotic arm attachment specifically designed for seamless ultrasound probe transitions that significantly reduce the amount of time required to switch probes. It was engineered with a triple rack and dual pinion system (Figure S1) that ensures efficient and synchronized movements of both ultrasound probes (Fig. 2, Row 1). When one probe is elevated into its operational position, the other is simultaneously and smoothly retracted into a standby position (Fig. 2, Row 1).

The device was attached to the 2F-140 adaptive gripper, which was used to control the third rack and pinion. The opening and closing motion of the gripper slides the rack left or right while rotating the pinion clockwise or counterclockwise. The rotation of this pinion controls the rotation of the pinion attached on the opposite side. This controls the vertical motion of the two probe-holding racks. Therefore, probes are exchanged by opening or closing the gripper. The operations orchestrated by the subtle actions of the gripper eliminate the need for manual interchange and reduce the time required for transition. The device was manufactured with resin (Formlabs, Somerville, MA, USA) by additive manufacturing.

#### Modular design

The Modular Design can swap ultrasound probes through rapid docking and undocking of each probe module. The prototype was fabricated with PLA (Raise3D, Irvine, CA, USA) and resin (Formlabs, Somerville, MA, USA) by additive manufacturing. Each probe was secured within a modular enclosure with a design key at the top surface of the device as well as a design key on either side of the device (Figure S2). This modular probe adapter was then positioned within a docking station affixed to the operational surface.

For procedure initialization, the receiving end part was integrated with the robotic arm allowing for the robotic arm to lower down and receive the top keyed end of the modular probe adapter. The robotic arm fitted with the end receiver then rotates around the keyed module, held stationary by the docking station, locking the modular adapter into place. At this stage, the robotic arm can lift the module with the probe.

For swapping between transducers, the module is lowered to the docking station by the robotic arm (Fig. 2, Row 2), lining up the side keys with the slots of the dock. The robotic arm’s twist motion is then reversed until the module’s side keys are locked into the docking station slots. Continued rotation releases the module’s top key from the robotic arm’s end receiver. Once released, the robotic arm is detached from the probe module. The robotic arm can then perform the reverse operation for attaching to a different transducer module (Fig. 2, Row 2).

#### Dual-end design

The Dual-End Design was engineered for probe models with scanning functionality available on either end of the transducer. In its current form, the design used the Vscan Air™ CL, a commercially available wireless dual-ended model featuring Wi-Fi and Bluetooth capabilities, with linear and curvilinear probes on each end. The model’s wireless feature overcomes the limitation imposed by traditional wired models which potentially limit the robotic arm’s range of motion during operation.

The probe adapter was designed to rotate the ultrasound probe in place and lock at two set positions for application of either the curvilinear or linear end of the probe (Figure S3). The robotic arm was programmed to exchange probe ends by rotating the probe adapter 180° around a static bar. This programmed method of exchange bypasses the need for additional motorized features and makes use of the probe’s wireless benefits (Fig. 2, Row 3). While rotating from its starting position, four spring-loaded steel balls recess into the holder and allow the Vscan Air™ to find its next set position. When the holder reaches one of the endpoint positions, the spring-loaded balls will home into its divot, and lock the ultrasound probe in place. The prototype was fabricated with PLA (Raise3D, Irvine, CA, USA) and resin (Formlabs, Somerville, MA, USA) by additive manufacturing.

### Testing descriptions

Two different testing configurations were used to evaluate the performance of the varied probe holder designs. The eFAST testing configuration used a torso mannequin which allowed the US probe to be positioned at the proper eFAST scan sites. However, this testing setup was not ultrasound compliant, so a second testing setup was configured which allowed the robotic arm to capture ultrasound images of the femoral artery region of a US compliant tissue phantom. These two testing modalities are detailed below.

#### eFAST testing

**Figure 3:**
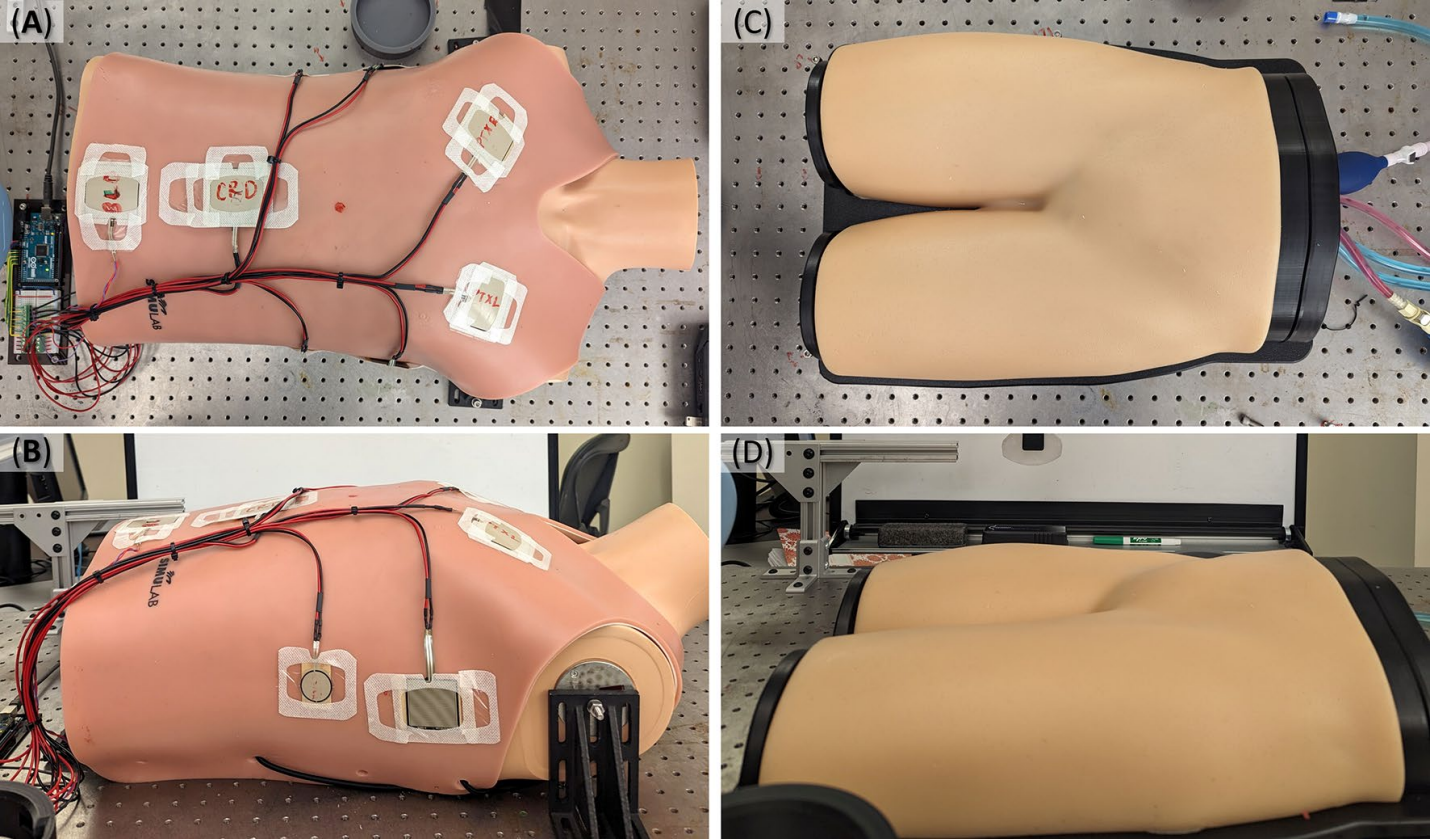
Test platforms for evaluating probe adapters. Torso mannequin with force sensing resistor configuration for testing repeatability of full eFAST exam; shown from (**A**) top view and (**B**) side view. Ultrasound tissue phantom of femoral region used for image repeatability testing; shown from (**C**) top view and (**D**) side view.

A torso mannequin (Amazon, Seattle, WA, USA) was fitted with a silicone eFAST vest (Simulab, Seattle, WA) situated with force sensing resistors (SparkFun Electronics, Niwot, CO, USA) approximately placed at each of the eFAST scan points (Fig. 3A, B). For each probe adapter design, the robot was programmed to complete an eFAST exam on the torso mannequin. Robotic arm movements were controlled by specifying the coordinates for the desired position of the robotic arm, which would place the probe at the desired contact site of the body. The robotic arm was then programmed to continue moving towards the body until a collision was detected and contact was made. The variation in geometry of each design required slight variations in the path of travel; however, each probe design was programmed to achieve contact with the body and apply force at each scan point. Each test was conducted five times (n = 5) with both the curvilinear and linear probes for a total of ten trials conducted per each of the four probe adapter approaches.

Each test began with the probe adapter and US probe attached to the robotic arm. Through programmed robotic movements, the probe scanned each eFAST scan point, and repeated successful contact with the scan site was determined by force sensing resistors placed at each scan site (Fig. 6, left diagram). Before and after the eFAST scan, the UR5e was programmed to have the US probe make contact with a force sensor (Mark-10, Copiague, NY, USA). The UR5e was programmed to repeat each scan with precise positions specified so that any difference in force measurements by the force sensor provided an indication of the repeatability of the application of force by each adapter design. Any change in the position or orientation of the probe within the adapter would alter the applied force. Therefore, the repeatability of force application provided a metric for evaluating the stability or security of the probe within the adapter. Finally, probes were exchanged at the end of each test to evaluate reliability of the probe swapping capability of each design. Additional tests (n = 5) were conducted for each design to determine the time required to swap probes alone.

#### Ultrasound image capture

Additional tests were conducted using a Femoral Vascular Access Training Model (Fig. 5 C, D) (CAE Healthcare, Montreal, Canada) to evaluate the repeatability of image capture by the robotic system. This model was used as it is an US compliant material with a vasculature that allows for assessing US image quality at different angles and applied forces. In the first round of testing, the UR5e robot was programmed to scan the leg phantom using a curvilinear probe orthogonally to the surface of the right leg using the three probe adapter designs and the Robotiq adaptive gripper. For comparison, images were also captured manually by a single human operator. The probe was then tilted ± 20° in four directions: cranial-caudal and medial–lateral (Figure S4). For each scanning technique (robotic and manual) ultrasound images of the phantom leg were captured using the Terason US machine, and additional images were captured by the Vscan Air™ wireless US device with the Dual-End Design.

The simulated vein, artery, and/or nerve features remained in view with each of the probe orientations and scanning approaches, to be able to track anatomical features across trials for evaluating image capture repeatability. Analysis was performed using MATLAB 2022b Image Labeler toolbox by pixel labeling. Each US image was labeled by three subjects to reduce labeling bias. The x and y centroid coordinates of each image label were calculated and compared across each design to evaluate the scanning repeatability of each design.

**Figure 4:**
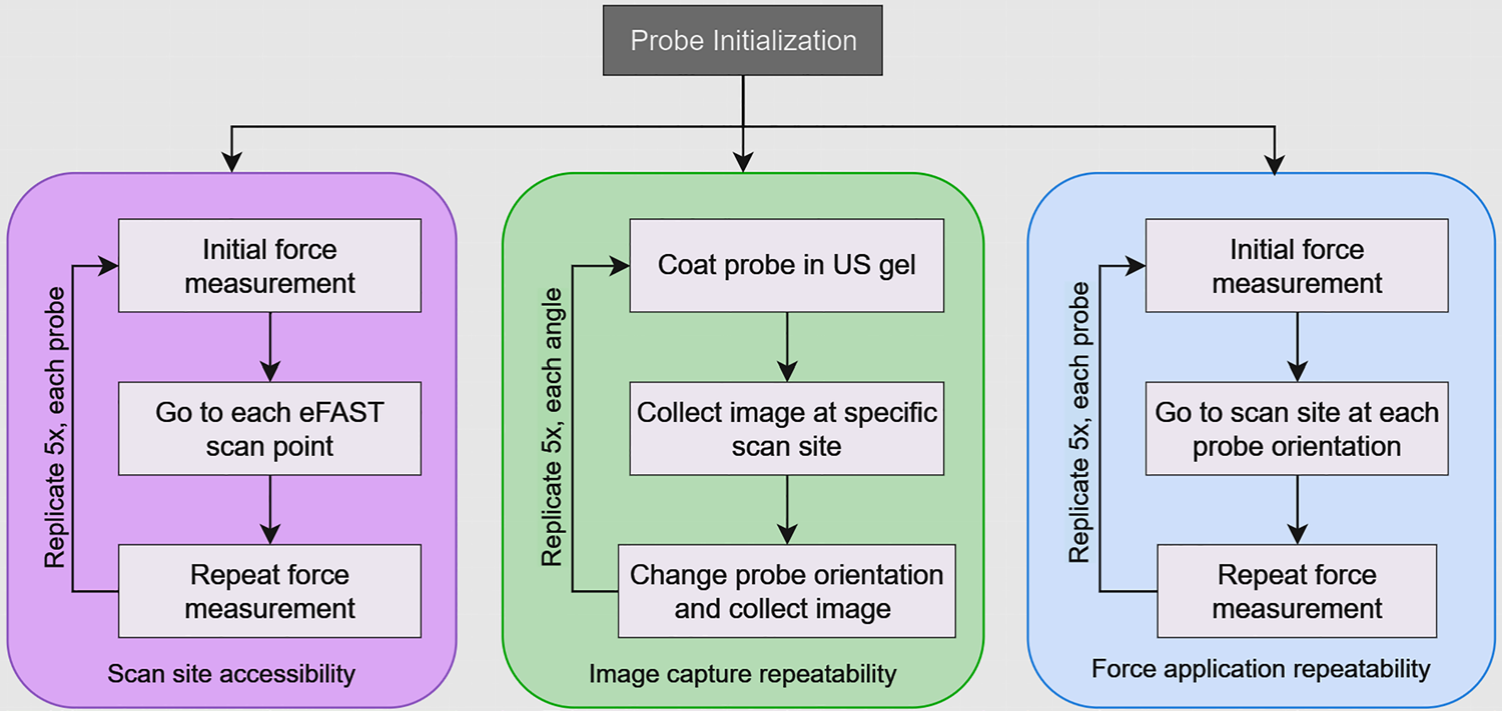
Flowchart of different testing regimens (from left to right) Scan site accessibility was determined with the eFAST torso model based on contact with force sensors at each scan site; image capture repeatability was measured with ultrasound compliant femoral region phantom and measured the variability in image capture by each design; force application repeatability was quantified before and after enough force was applied to acquire US images at different angles to assess if the US probe was properly secured in each probe adapter design.

Additional tests (n = 5) were performed without image capture to evaluate force application repeatability using a force sensor (Fig. 4, Right) (Mark-10, Copiague, NY, USA). In these tests, the curvilinear probe was also exchanged for the linear probe to further assess reliability of probe exchange by each design.

#### Statistical analysis

All statistical analyses were performed with GraphPad Prism 10.1.2 (La Jolla, CA, USA). To evaluate significant differences between the different US probe adapters, one-way analysis of variance (ANOVA) models were used. This was done for force repeatability and probe swap times to compare the four probe adapters. For the image capture repeatability analysis, a repeated measures ANOVA analysis was used where the five different probe positions were treated as repeated measures for each probe adapter as well as manual image acquisition. Each of these used a post-hoc Tukey’s test to measure significance wherein the significance threshold was taken as p = 0.05 for all analyses. ANOVA Post-hoc Šídák test was used when comparing specific groups such as evaluating the effect of US device (VScan Air™ or Terason) on image repeatability. Further, outlier exclusion criteria was used to remove data points using the ROUT method (robust regression followed by outlier identification)^27^ with a false discovery rate set at 1%.

## Results

### Probe adapter performance results

**Figure 5:**
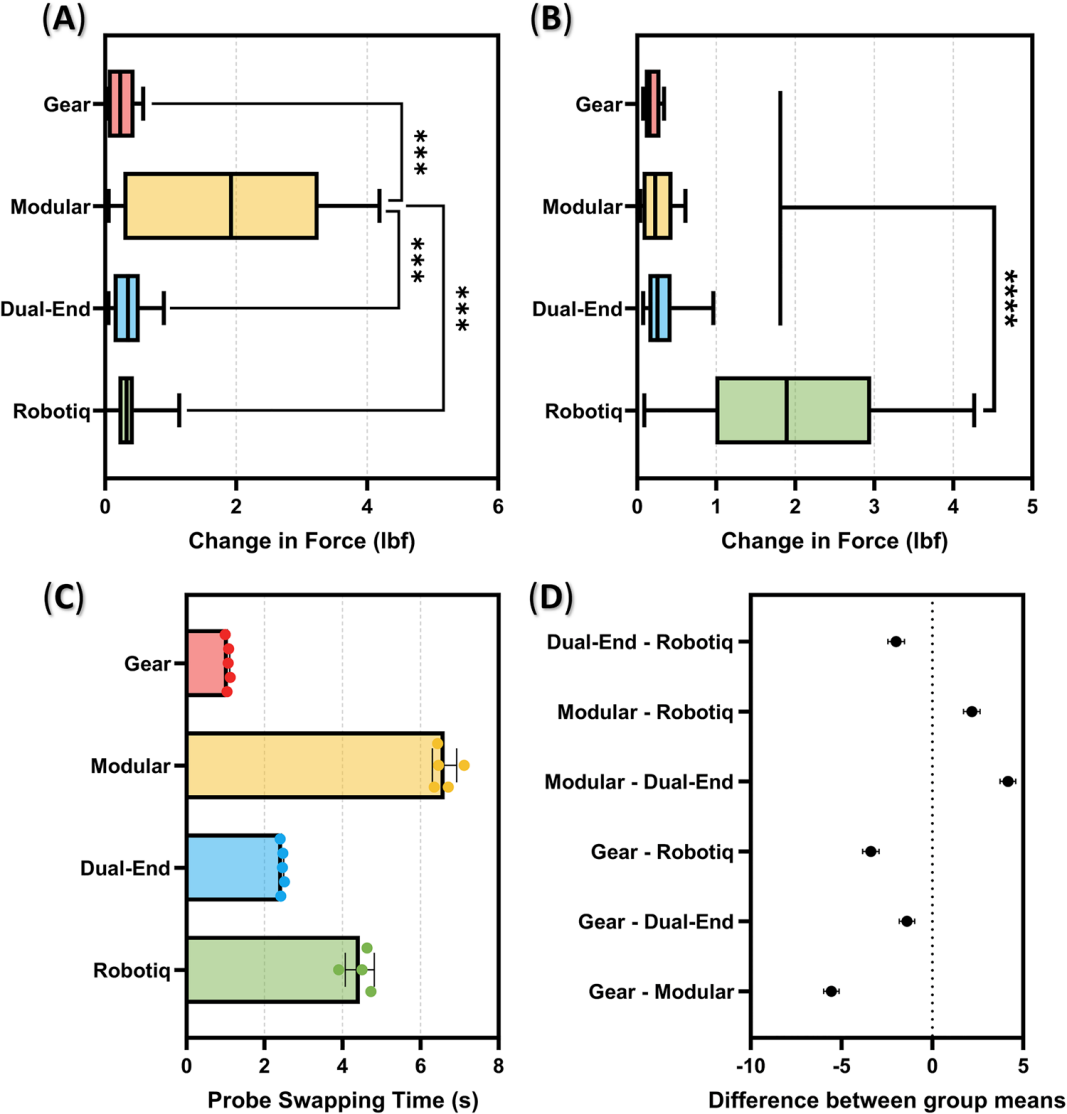
Force repeatability and probe swapping time results for each probe adapter. (**A, B**) Absolute difference between repeated force measurement for each probe adapter (n = 10) for the (**A**) torso and (**B**) phantom model. Statistically significant differences between two groups are denoted by asterisk. Box and whisker plots show the mean value and minimum and maximum values as the error bars. (**C**) Time required to swap probes between curvilinear and linear (n = 5). Error bars denote standard deviation. (**D**) 95% confidence intervals for the differences between each swap time group mean. As shown by confidence intervals not crossing 0, comparisons between each probe adapter were statistically significant.

The eFAST torso test setup allowed for assessment of each probe adapter's performance across each scan point as well as swapping between each US probe type. Starting with overall success at swapping probes and reaching scan points, all probe adapter designs were able to initialize and swap ultrasound transducers with a 100% success rate and access the scan points on the torso and phantom models with a similar high success rate. Next, we evaluated the variability of applied force by each adapter design, as the probe swapping required for an eFAST scan could result in probe placement error. For the torso mannequin tests, the applied force of each probe adapter design was repeatable for all but the Modular Design, with each other design having a significant difference vs. the Modular Design (Fig. 5A). Further, the variability for the Modular Design was much higher compared to the other probe adapters. Conversely, for the phantom testing, the Robotiq gripper had the highest force measurement error, with each other probe design having a significant difference compared to the Robotiq gripper (Fig. 5B).

The last metric evaluated for the torso phantom test was the length of time to swap probes. The Gear Design required 1.1 s compared to 2.4 s for the Dual-End Design and 6.6 s for the Modular Design (Fig. 5C). Meanwhile, manual exchange of probes required an average time of 4.4 s using the Robotiq gripper. All differences between probe adapters were statistically significant for time to swap probes (Fig. 5D).

### Probe adapter imaging results

A set of 5 ultrasound images of the phantom model were captured at a location where artery and vein features were evident with each of the probe adapters. The 5 images were taken at a repeated location with the probe orthogonal to the surface of the leg and then tilted ± 20° in the cranial-caudal and medial–lateral directions (Figure S4).

**Figure 6:**
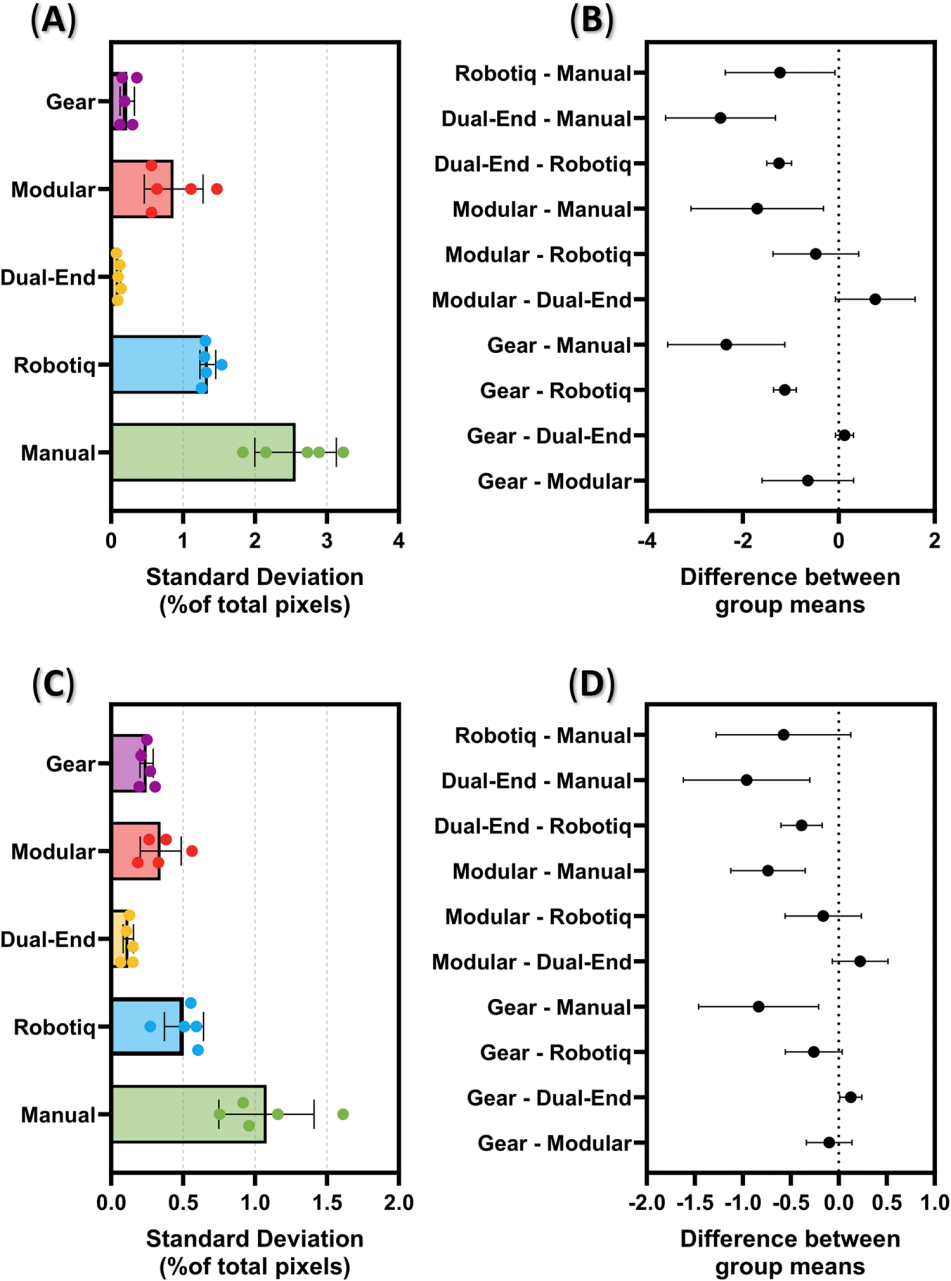
Ultrasound image repeatability results for each probe adapter. Results are shown for (**A, B**) the x-coordinate and (**C, D**) the y-coordinate of the measured feature centroid (N = 5). (**A, C**) Average standard deviation for each probe adapter and manual image acquisition. Error bars denote standard deviation for the 5 probe orientations used. (**C, D**) 95% confidence intervals for the differences between each swap time group mean. Statistically significant comparisons are denoted by confidence intervals not crossing 0.

Image variability was captured by tracking X and Y centroid coordinates of labeled features in images. Error in feature centroid coordinates were measures as standard deviation as a percent of total pixels in both the X and Y direction. Overall, manual image acquisition demonstrated the highest standard deviation at 2.6% and 1.1% for the X and Y coordinates, respectively (Fig. 6A,C). Next highest standard deviation values were with the Robotiq gripper at approximately 1.3% in the X direction and 0.51% in the Y direction. Of the probe adapter designs, the Modular Design performed the worst with standard deviation values reaching above 0.87% for the X direction and below 0.34% for the Y direction. Conversely, the Dual-End Design resulted in the lowest standard deviation at 0.10% and 0.12% for the X and Y coordinates, respectively. Differences between a number of the probe adapter and manual image capture pairings were statistically significant for both the X and Y directions (Fig. 6B,D).

**Figure 7:**
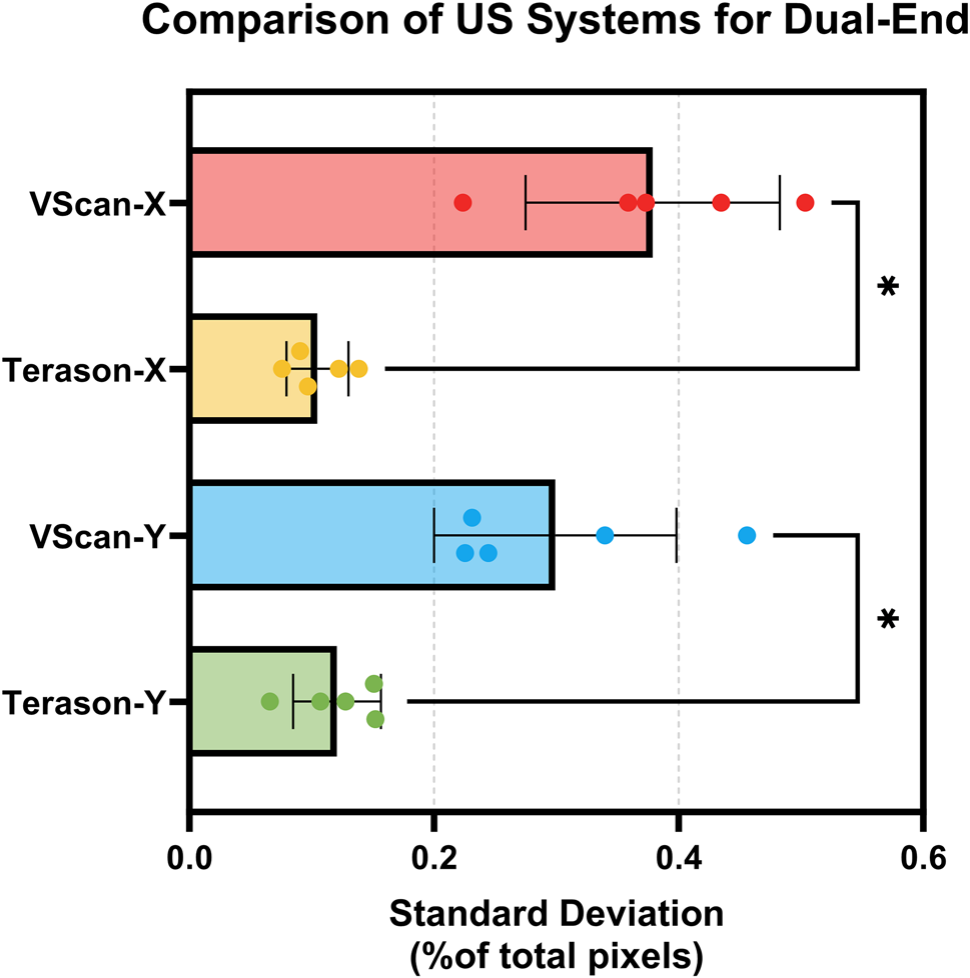
Comparison of ultrasound systems on the Dual-End Probe adapter. Comparison of average standard deviation for both the centroid X- and Y-coordinates for the VScan and Terason system are shown. Error bars denote standard deviation for the 5 probe orientations used. Differences between the US systems were statistically significance for both centroid coordinates (**p < 0.01, ANOVA post-hoc Šídák test).

These results with the Dual-End Design were captured with the Terason US system. Additional images for this probe adapter design were taken using the curvilinear side of the Vscan Air™ US probe. This introduced additional variability likely due to the Vscan Air™ software’s image capturing features. Instead of capturing single images a short 1-s video was captured by the Vscan Air™ software. The last frame of the video was exported and used to evaluate image repeatability. Images captured by the Dual-End Design using the Vscan Air™ probe resulted in statistically significant differences in both directions, with standard deviation of 0.38% and 0.30% for the X and Y coordinates, far exceeding the 0.10% X and 0.12% Y values using the Terason system (Fig. 7).

### Overall performance summary

For each test, the end-adapters were scored so that the best performing design received a score of 100%, and the worst performing received a score of 0%. The remaining two designs received a calculated score that was normalized based on their performance relative to the results of the best and worst performing designs. This was done across the five performance measures for each end-adapter design. The Gear Design was best performing and thus received an overall score of 100%. However, the Dual-End Design scored similarly with a result of 96%. The Modular Design performed worse at a score of 50%, comparable to the Robotiq gripper at a score of 48%.

## Discussion

Ultrasound imaging is an essential tool for emergency triage in order to allocate limited resources to severely injured casualties especially during remote medical situations such as those faced in combat casualty care. This need is expected to rise on the future battlefield where peer and near-peer adversaries will contest airspace making medical evacuations even more limited. Unfortunately, US imaging is only a viable triage tool if proper images can be captured, which is not guaranteed in high stress situations or by non-subject matter expert ultrasonographers. The robotic platform detailed here can automate image acquisition for this application if developed for reliable US image collection.

Overall, all end-adapter designs were able to successfully reach the eFAST scan points and demonstrated a high rate of successful probe exchange. Differences between the designs were evident across other testing criteria, such as application force, where we demonstrated that encasing the probe provided more reliable force application than by using the Robotiq gripper alone. The Gear Design and the Dual-End Design both provided a high degree of image repeatability with low variability of 0.24% and 0.11%, respectively, and outperformed other probe adapters, highlighting these as the highest performing across all evaluated criteria. The difference in performance between the Gear Design and the Dual-End Design was minimal, with both designs providing quick probe exchange times. For the Gear Design, size may limit its ability to reach a wider range of scan points in variable testing configurations. The Dual-End Design was intended for use with wireless US probe systems, having limited functionality for wired probes, with cables that would impede the 180-degree rotations required to swap probe types. Future improvements will include modifications to address these challenges for both end-adapter designs.

The Modular Design performed similarly to the Robotiq gripper, due to the additional time required to exchange probes and lower performance on image repeatability, relative to the other designs. The Modular Design’s worse performance across the evaluated testing criteria was likely due to the greater degree of physical exchange required by the modular components compared to the other more compact designs. However, the Modular end-adapter provided additional functionality through the ability to swap beyond just two probe styles and may even be further developed to support additional attachments for therapeutic interventions, such as needle decompression for relieving tension pneumothorax injury^4^.

While the use of a single tissue phantom torso was an initial step within the scope of this project, future research for robust performance will include using a more diverse set of anatomical models, accounting for subject variability. This would be key for further improvements to the end-adapter designs, as proper access to all eFAST scan points in a wide array of human torsos would be essential for an automated image acquisition platform. As mentioned earlier, the ultrasound image capture repeatability for each end-adapter was assessed using a femoral vascular access phantom. The study design using this model is limited by comparison to a single human operator capturing manual US images. Images captured by a greater pool of ultrasound technicians would provide a more accurate accounting of manual image collection. Furthermore, a wider range of scan data may improve understanding of the range of techniques an ultrasound technician may apply, such as altering the transducer angle and adjusting the force applied to the body to acquire a proper US image.

Future testing will evaluate the ability of the robotic arm with probe holder attachments to access and capture quality images of relevant features at the proper eFAST scan points using a more robust model. Our group has developed an eFAST tissue phantom with positive and negative injury states at each scan site of the exam, that will allow to assess performance for the end-adapters while capturing eFAST relevant ultrasound images^8^. Future work will involve developing and testing a system for replicating such techniques using reinforcement learning or similar approaches to ensure feasibility of proper image acquisition by the robotic arm and probe adapters.

## Conclusion

Our testing demonstrated that the robotic platform may be used to programmatically navigate to reach all the proper eFAST scan points. The end-adapter designs allowed for the robotic arm to hold an ultrasound probe in a reliable and repeatable manner. In future efforts, we will focus on integrating AI models with the robotic system so it can properly identify, access, and image eFAST scan points. Automated ultrasound image capture and interpretation may lower the skill threshold required for medical assessment and provide accessible higher-quality care for combat casualty care.

### Supplementary Information

Supplementary Information.

## Data Availability

The datasets presented in this article are not readily available because they have been collected and maintained in a government-controlled database that is located at the US Army Institute of Surgical Research. As such, this data can be made available through the development of a Cooperative Research & Development Agreement (CRADA) with the corresponding author. Requests to access the datasets should be directed to Eric Snider, eric.j.snider3.civ@health.mil.
