# Supplementary material for: Design and testing of ultrasound probe adapters for a robotic imaging platform
Source: Sci Rep. 2024 Mar 1;14:5102. doi: 10.1038/s41598-024-55480-0 (PMC10907673; doi:10.1038/s41598-024-55480-0)
Supplement: Supplementary file 1 — Supplementary Information. [file 41598_2024_55480_MOESM1_ESM.pdf]

Design and Testing of Ultrasound Probe Adapters for a Robotic Imaging Platform

Krysta-Lynn Amezcua[1], James Collier[1], Michael Lopez[1], Sofia I. Hernandez-Torres[1], Austin Ruiz[1], Rachel Gathright[1], Eric J. Snider[1*]

[1] Organ Support and Automation Technologies Group, U.S. Army Institute of Surgical Research, JBSA Fort Sam Houston, San Antonio, TX 78234, USA

* Corresponding Author: eric.j.snider3.civ@health.mil (E.J.S.)

Supplementary Information

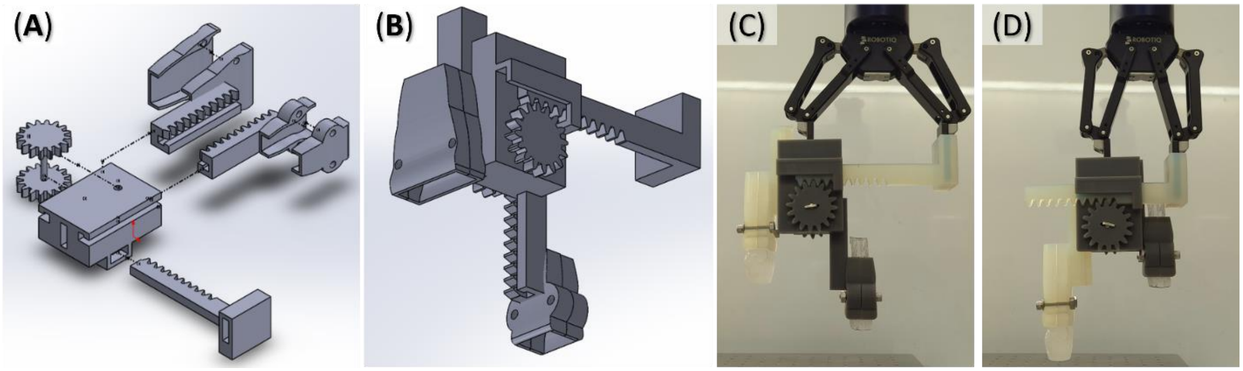

Figure S1. Overview of The Gear Design ultrasound probe holder. *Engineering diagram of The Gear Design in (A) exploded view and (B) assembled view. The design can switch between (C) linear and (D) curvilinear transducer configurations.*

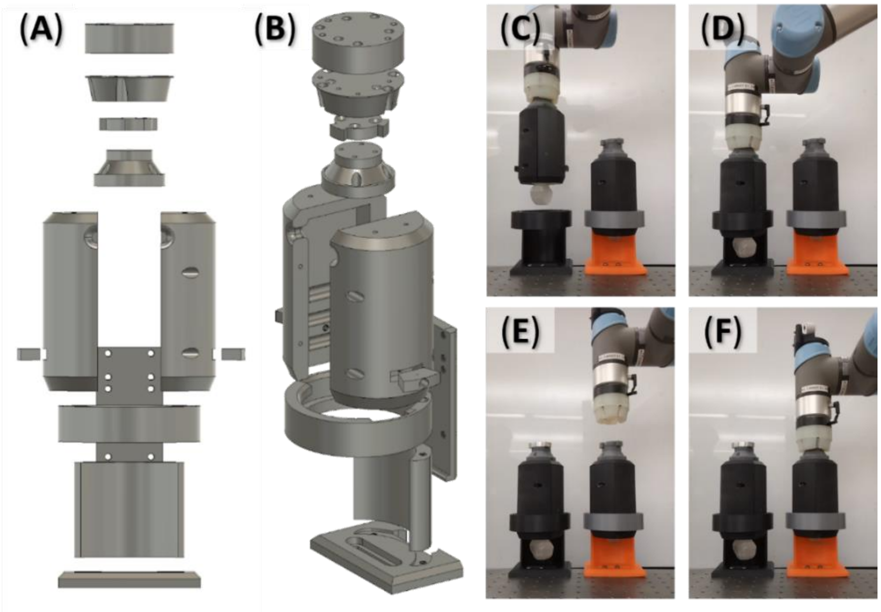

Figure S2. Overview of the Modular Design ultrasound probe holder. *Engineering diagram of the Modular Design in (A) front and (B) oblique exploded view. (C-F) The design can swap between linear and curvilinear transducer housed in docking stations.*

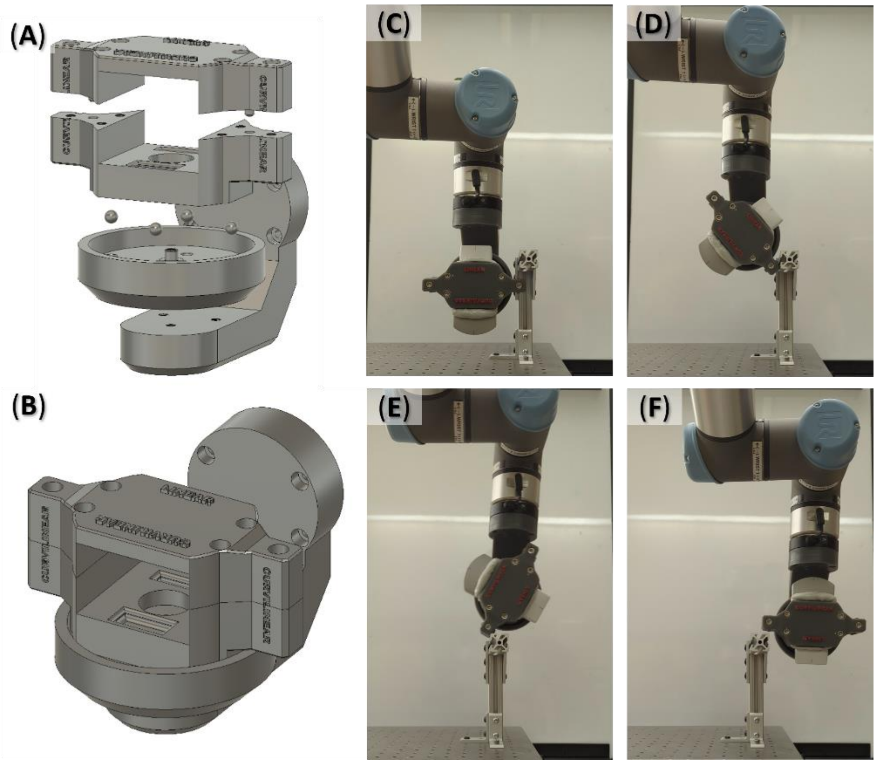

Figure S3. Overview of The Dual-End Design ultrasound probe holder. *Engineering diagram of The Dual-End Design in (A) exploded view and (B) assembled view. (C-F) The design can swap between linear and curvilinear transducer by rotation around a horizontal bar.*

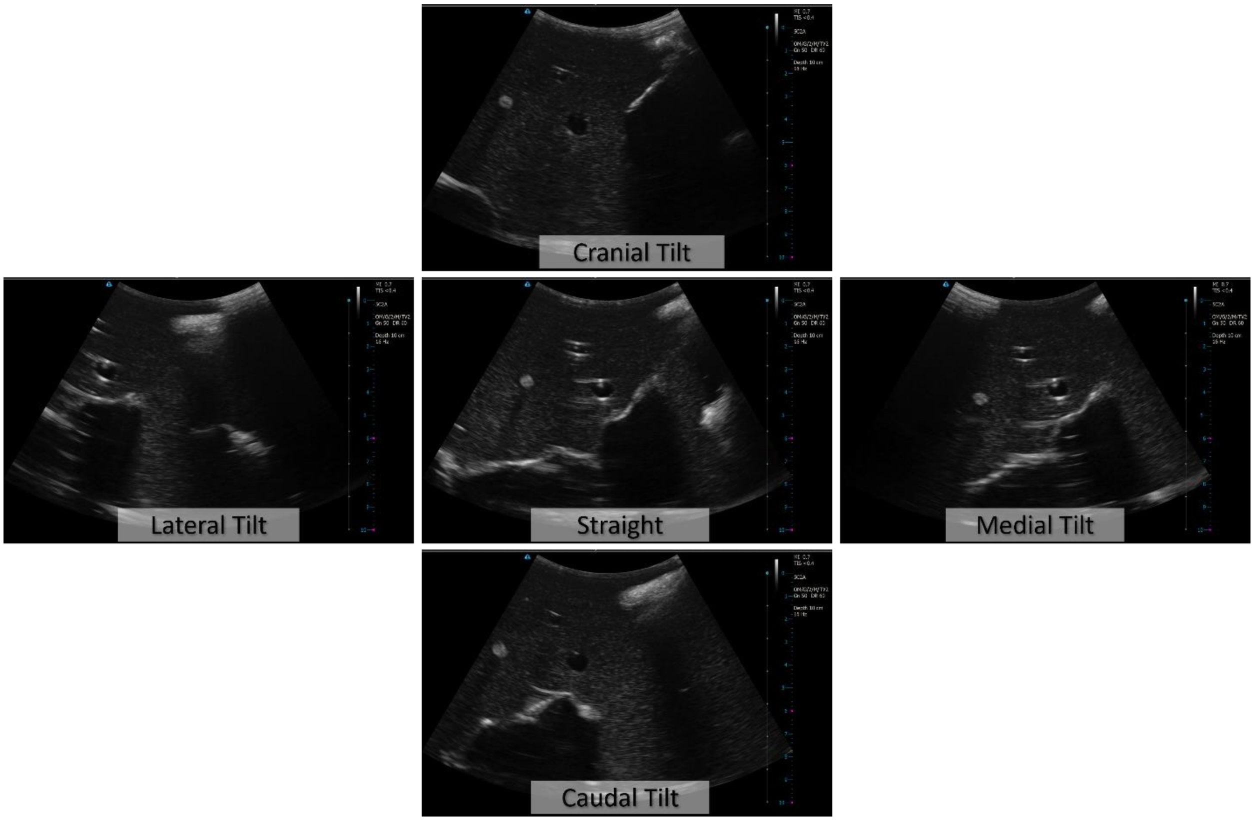

Figure S4. Representative ultrasound images from repeatability testing with tissue phantom. *US images of the phantom leg were captured to evaluate scanning repeatability of each probe adapter design at different angles (identified in figure).*
